# Organic Solvent-Free and Emulsion Self-Templating Synthesis of 3D Macroporous SiO*_x_*/C@C for Durable Lithium-Ion Battery Anodes

**DOI:** 10.3390/polym18111398

**Published:** 2026-06-04

**Authors:** Jianing Zong, Kaize Si, Jingjing Li, Xiaomei Wang, Xu Zhang

**Affiliations:** Hebei Key Laboratory of Functional Polymers, School of Chemical Engineering and Technology, Hebei University of Technology, Tianjin 300130, China

**Keywords:** lithium-ion battery, water-in-oil emulsion, free-radical polymerization, organic solvent-free synthesis, 3D macroporous structure, SiO*_x_* anodes

## Abstract

SiO*_x_* anodes are highly promising for next-generation lithium-ion batteries due to their superior theoretical capacity. However, issues such as drastic volume expansion and low initial Coulombic efficiency (ICE) impede their practical use. While macroporous architectures can mitigate these challenges, traditional fabrication often depends on tedious hard templating methods and significant organic solvent consumption. In this work, we report a sustainable, emulsion-self-templated and organic solvent-free strategy to synthesize a carbon-coated 3D macroporous SiO*_x_*/C composite (3DM-SiO*_x_*/C@C). Our approach uniquely integrates radical polymerization with a water-in-oil emulsion and sol–gel process, followed by chemical vapor deposition (CVD). The 3D macroporous framework is generated via in-situ emulsion droplets acting as self-templates, effectively eliminating the need for external sacrificial templates and toxic etchants. Notably, this organic solvent-free process achieves an exceptional precursor to (precursor + organic solvent) mass ratio of 1.0, contrasting sharply with conventional methods (0.0044–0.17). The resulting hierarchical structure, characterized by interconnected macropores and a uniform carbon coating, significantly enhances structural integrity and electronic conductivity. Electrochemical evaluations reveal that 3DM-SiO*_x_*/C@C exhibits an improved ICE of 74.32% and long-term cycling stability even at a high current density of 1.0 A g^−1^ compared to non-porous and uncoated counterparts. This integrated synthesis offers a green and scalable pathway for developing high-performance silicon-based anodes for large-scale energy storage.

## 1. Introduction

Global energy structure transformation has emerged as an urgent imperative to address environmental degradation and climate change, with the core pathway residing in the progressive replacement of traditional fossil fuels by renewable energy sources [1]. However, clean energy represented by solar and wind power exhibits pronounced geographical intermittency and uneven distribution [2,3]. Therefore, the advancement of high-performance energy storage technologies is a prerequisite for the widespread implementation of renewable energy sources.

Owing to their superior merits, such as high energy density, extended cycle life, and negligible memory effect, lithium-ion batteries (LIBs) have emerged as the preferred power source for electric vehicles, smart grids, and aerospace exploration [4,5]. Nevertheless, graphite-based anode materials—currently dominant in commercial LIBs—possess a theoretical specific capacity limited to 372 mAh g^−1^, severely restricting further enhancement of battery energy density [6]. Thus, developing novel anode material systems with superior lithium storage capabilities holds significant importance for advancing next-generation high-energy-density battery technologies.

In the pursuit of next-generation anode materials, although pure silicon (Si) anodes exhibit an exceptionally high theoretical capacity, their severe volume expansion (>300%) and the instability of the solid electrolyte interphase (SEI) layer during continuous lithiation/delithiation processes lead to rapid degradation of battery performance, severely hindering their practical application [7,8,9]. In contrast, silicon suboxide (SiO*_x_*) has emerged as a highly promising candidate for industrialization, distinguished by its cost-effectiveness, earth abundance, and a high theoretical specific capacity exceeding 2000 mAh g^−1^ [10,11,12,13,14]. During the initial lithiation process, the oxygen in SiO*_x_* reacts with lithium to form an inactive buffer matrix in situ (primarily consisting of Li_2_O and lithium silicates) [15,16]. This intrinsic matrix effectively cushions the structural stress, reducing the volumetric expansion of SiO*_x_* to approximately 200% [17,18]. However, despite this mitigated expansion, SiO*_x_* still suffers from severe mechanical degradation. The consequent mechanical delamination between electrode materials and current collectors, structural pulverization, and continuous reconstruction of the SEI directly lead to rapid capacity decay and reduced cycle life [19,20,21]. Furthermore, the intrinsically low electrical conductivity of SiO*_x_* (<10^−5^ S cm^−1^) severely restricts internal electron transport kinetics, further aggravating electrode polarization phenomena [22]. Addressing the synergistic enhancement of structural stability and electrochemical performance in SiO*_x_*-based anodes during long-term cycling remains a key scientific challenge impeding their commercial application.

Recent research advancements demonstrate that constructing macroporous-structured SiO*_x_* materials with carbon doping can significantly improve their electrochemical performance as lithium-ion battery anodes [23,24,25,26,27]. Specifically, the mechanisms primarily include: (1) The porous structure provides effective buffering voids for accommodating the volumetric expansion of SiO*_x_* during lithiation/delithiation, thereby suppressing material pulverization and structural collapse [28,29]. (2) The high specific surface area of the porous structure substantially shortens the solid-state diffusion path of lithium ions, while its channels facilitate electrolyte infiltration [23,30]. (3) The introduction of a carbon matrix enhances the electrical conductivity and promotes the formation of a uniform and stable SEI film [31]. However, existing synthesis strategies still face critical challenges: (1) The excessive consumption of organic solvents during synthesis increases costs, energy consumption, and causes environmental pollution. (2) Porous materials typically synthesized via soft/hard templating methods (e.g., PS/SiO_2_ microspheres or Al) involve multi-step processes including template preparation and removal, which are complex and wasteful. Etching agents (strong alkaline or acidic solutions) are often hazardous. A systematic comparison of various templating strategies, highlighting their usage, template removal steps, and scalability, is summarized in Table S2 [23,32,33,34,35,36,37,38]. (3) High specific surface area SiO*_x_* materials frequently undergo excessive side reactions during initial cycles, including massive consumption of active electrolyte components and formation of unstable SEI films, resulting in low ICE [39,40]. These issues impede the commercial application of SiO*_x_* materials.

In this work, we develop an emulsion self-templated and organic solvent-free method to construct carbon-coated 3D macroporous SiO*_x_*/C (3DM-SiO*_x_*/C@C). Herein, the term “organic solvent-free” specifically denotes the complete exclusion of conventional, non-reactive volatile organic media (such as ethanol, acetone, or DMF) that function purely for dispersion or dissolution. The method involves the synthesis of poly(vinyltrimethoxysilane) (PVTMS) by radical polymerization, the formation of a W/O emulsion system with PVTMS and water, the production of 3DM polysilsesquioxane (PSSQ) by a sol–gel method, and carbon-coating via chemical vapor deposition (CVD) processes. The obtained macroporous structure effectively buffers volume variations during charge/discharge cycles, maintaining the structural stability of the electrode material. Furthermore, this 3D macroporous architecture facilitates highly efficient electron and ion transport within the electrode, providing robust support for higher mass loading. Meanwhile, the synergistic effect of carbon doping and carbon coating induces the formation of a stable SEI film, thereby suppressing the continuous consumption of active materials during cycling. The 3DM-SiO*_x_*/C@C electrode demonstrates outstanding electrochemical performance: an initial discharge capacity of 1615 mAh g^−1^ with a high ICE of 74.32%, retaining 515.9 mAh g^−1^ after 500 cycles at a current density of 1.0 A g^−1^, corresponding to a capacity retention rate of 71.4%. This work provides a potentially scalable preparation method for the 3DM-SiO*_x_*/C@C anode for improved electrochemical performance in lithium-ion batteries.

## 2. Materials and Methods

### 2.1. Chemicals

Vinyltrimethoxysilane (VTMS, 98%, Aladdin Industrial Corporation, Shanghai, China), di-tert-butyl peroxide (DTBP, 97%, Shanghai Macklin Biochemical Co., Ltd., Shanghai, China), Span 80 (Aladdin Industrial Corporation), polyglycerol polyricinoleate (PGPR, the contents of diglycerides, triglycerides, and tetraglycerides ≥ 75 wt%, while the contents of heptaglycerides and higher polymers ≤ 10 wt%, Shanghai Macklin Biochemical Co., Ltd.), ammonia solution (NH_3_·H_2_O, 25 wt%, Aladdin Industrial Corporation), acetylene black (CP, Denka Co., Ltd., Tokyo, Japan), sodium alginate (CP, Beijing Solarbio Science & Technology Co., Ltd., Beijing, China), and electrolyte (Kolod Chemical Technology (Shanghai) Co., Ltd., Shanghai, China) were used as received without purification.

### 2.2. Synthesis of 3DM-SiO_x_/C@C

The preparation of carbon-coated porous SiO*_x_* (3DM-SiO*_x_*/C@C) involved free-radical bulk polymerization, sol–gel processing, and chemical vapor deposition (CVD) techniques. First, oligomer PVTMS was synthesized by bulk radical polymerization according to a previous report [41]. This specific protocol was selected to synthesize an oligomer with a suitable viscosity, which is crucial for the subsequent emulsion process. Specifically, initiator DTBP (0.169 g) was dissolved in the reactive organosilicon monomer VTMS (14.8 g). The resulting solution was transferred into a 1000 mL four-neck flask and subsequently polymerized at 120 °C with stirring at 350 rpm for 48 h under an argon atmosphere (flow rate: 30 mL min^−1^). A reaction time of 48 h was specifically chosen to ensure complete polymerization and obtain PVTMS with a suitable viscosity, yielding a colorless, viscous liquid. Subsequently, a W/O emulsion system was constructed: Span 80 (0.16 g) and PGPR (0.32 g) were dissolved in PVTMS (4 g), then homogenized via 10,000 rpm high-speed shear dispersion for 2 min to form a uniform oil phase. Ammonia solution (1.8 g, containing 0.8 g of 25 wt% NH_3_·H_2_O and 1.0 g of deionized water) was added dropwise at a constant rate of 0.6 mL min^−1^ at room temperature, followed by 2 min continuous high-speed stirring to complete emulsification. After 24 h aging at room temperature, the emulsion formed a polysilsesquioxane with three-dimensional macropores (3DM-PSSQ)/H_2_O composite gel. The obtained wet 3DM-PSSQ precursor was first pre-frozen at −20 °C overnight. Subsequently, the sample was transferred to a freeze-dryer and lyophilized at −50 °C for 24 h to thoroughly remove the internal solvent while preserving the macroporous architecture. The resulting dried 3DM-PSSQ was pyrolyzed at 900 °C for 3 h under argon (heating rate: 3 °C min^−1^) to obtain the porous substrate (3DM-SiO*_x_*/C), followed by CVD surface modification using an Ar/C_2_H_2_ mixed gas (volume ratio = 0.9:0.1) for 40 min to yield the final 3DM-SiO*_x_*/C@C composite, which contains approximately 0.1549 g of CVD-deposited carbon per 1 g of the final product. For comparison, two distinct control samples were deliberately designed: (1) a porous SiO*_x_* sample without CVD treatment (3DM-SiO*_x_*/C) was prepared to verify the protective effect of the CVD carbon coating, and (2) a carbon-coated non-porous SiO*_x_* sample obtained by direct sol–gel synthesis of VTMS without the emulsion template (BK-SiO*_x_*/C@C) was prepared to verify the structural buffering effect of the 3D macropores. To avoid destroying the 3D macroporous structure through over-pulverization, the bulk samples were first gently crushed into 1~2 mm fragments using an agate mortar and pestle, followed by careful grinding directly on a 400-mesh screen (aperture 38 μm). Particles smaller than 38 μm immediately passed through the screen, thereby preventing excessive damage to the macropores. The final powder yield after this crushing and sieving process was approximately 90%.

### 2.3. Material Characterizations

Scanning electron microscope (SEM, FEI Nova NanoSEM450, FEI Company, Hillsboro, OR, USA) was employed to characterize the morphology and elemental distribution of the samples. Transmission electron microscopy (TEM, FEI Nova Talos F200S, FEI Company, Hillsboro, OR, USA) was employed to characterize the microscopic morphology and the carbon coating layer of the materials. X-ray diffraction (XRD) patterns were recorded by a Bruker D8-Davinci instrument (Bruker, Karlsruhe, Germany) with Cu Kα radiation (40 kV, 150 mA) at a scan rate of 6° min^−1^ and the measured scan range was set to 10~80°. Fourier transform infrared (FT-IR) spectra in the range of 400 to 4000 cm^−1^ were recorded using a Bruker VECTOR-22 FT-IR spectrometer (Bruker, Ettlingen, Germany). The Raman spectra of samples were obtained within the wavelength range of 750~2000 cm^−1^ using a Renishaw inVia Reflex UV Raman spectrometer (Renishaw plc, Wotton-under-Edge, UK). The surface chemical states of the samples were analyzed by an XPS instrument (Thermo ESCALAB-250Xi, Thermo Fisher Scientific, Waltham, MA, USA). Gel permeation chromatography (GPC, Agilent 1260 Infinity II, Agilent Technologies, Santa Clara, CA, USA) was performed to determine the molecular weight of the polymers. Thermogravimetric analysis (TGA) was conducted using a TA Instruments SDT-Q600 analyzer (TA Instruments, New Castle, DE, USA) from room temperature to 900 °C at a heating rate of 10 °C min^−1^ under an air flow of 100 mL min^−1^. Nitrogen adsorption–desorption isotherms were measured at 77 K using a Micromeritics ASAP 2460 (Micromeritics Instrument Corporation, Norcross, GA, USA) surface area and porosity analyzer. Prior to the measurements, the samples were degassed under vacuum at 200 °C.

### 2.4. Electrochemical Characterizations

The electrochemical performance was evaluated using CR2032 coin-type half-cells, which were assembled as follows: first, the prepared SiO*_x_* samples (75 wt%), sodium alginate (15 wt%), and acetylene black (10 wt%) were mixed with an appropriate volume of deionized water to form a homogeneous slurry, which was then coated onto copper foil. After drying at 80 °C, the coated foil was punched into circular electrode disks with a diameter of 12 mm. The mass loading of the active material was controlled at approximately 1.0 mg cm^−2^. This specific loading was selected to accurately evaluate the intrinsic electrochemical performance of the designed materials, preventing the sluggish ion/electron transport kinetics associated with overly thick electrodes from affecting the measurement of intrinsic properties.

The electrolyte consisted of 1 M LiPF_6_ dissolved in a mixture of ethylene carbonate (EC), ethyl methyl carbonate (EMC), and dimethyl carbonate (DMC) (1:1:1, *v*/*v*/*v*). To ensure sufficient wetting of the separator and electrodes, as well as to completely fill the inherent macroscopic dead volume of the CR2032 coin cell architecture, an initial volume of 75 μL of electrolyte was injected into each cell. Although this yields an apparent electrolyte-to-active material ratio of approximately 62.5 μL mg^−1^, which is higher than commercial lean-electrolyte standards, this flooded protocol is essential for laboratory-scale fundamental testing. This assembly strategy ensures that the internal macroscopic void space is completely saturated, thereby minimizing cell-to-cell variations and guaranteeing the reproducibility of the intrinsic electrochemical data. Cell assembly was performed in an argon-filled glove box (Super (1220/750/900), Mikrouna, Shanghai, China), employing Celgard 2500 polypropylene separators and lithium metal counter electrodes. Galvanostatic charge/discharge (GCD) profiles were recorded using a LAND 2001A testing system (Wuhan LAND Electronics Co., Ltd., Wuhan, China) within a voltage window of 0.01~3.0 V. Prior to long-term cycling at 1.0 A g^−1^, three activation cycles were conducted at 0.1 A g^−1^. Electrochemical impedance spectroscopy (EIS) and cyclic voltammetry (CV) measurements were conducted on a CHI650B electrochemical workstation. The CV scan covered a voltage range of 0.01 to 3 V (vs. Li/Li^+^), while the EIS frequency scan spanned 0.01 Hz to 100 kHz. Lithium-ion diffusion coefficients were determined by the galvanostatic intermittent titration technique (GITT) using a pulse current density of 0.1 mA g^−1^ with pulse/relaxation durations of 15 min and 2 h, respectively.

## 3. Results and Discussion

### 3.1. Structural Characterizations

#### 3.1.1. Synthesis Mechanism and Chemical Transformation

**Figure 1:**
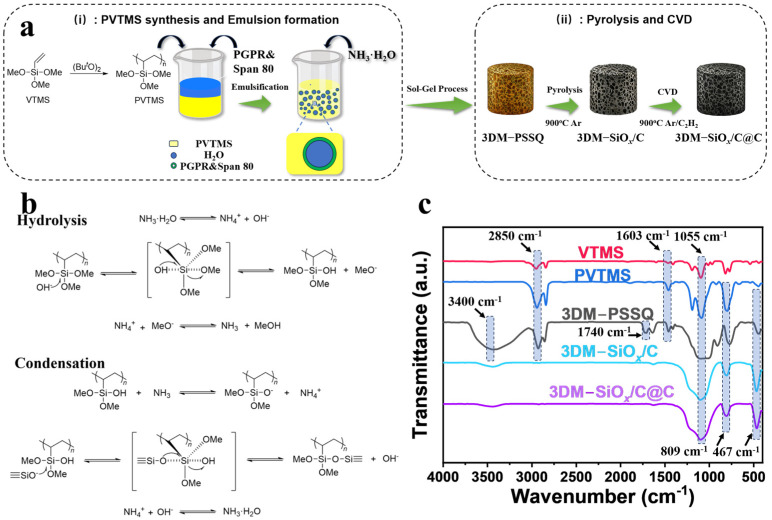
(**a**) Schematic illustration of the synthesis of the 3DM-SiO*_x_*/C@C electrode. (**b**) The mechanism of hydrolysis and condensation of PVTMS to form 3DM-PSSQ under the catalysis of ammonia. (**c**) FT-IR spectra of representative products obtained at each stage.

The preparation process of 3DM-SiO*_x_*/C@C is illustrated in Figure 1a. First, oligomer PVTMS was synthesized via radical bulk polymerization of VTMS using DTBP as the initiator. The resulting PVTMS product is a viscous oligomeric liquid. The molar mass and its dispersity were characterized by GPC (Figure S1 and Table S1, M_w_ = 4976 and MWD = 1.85). Subsequently, Span 80 and PGPR were selected as composite emulsifiers to form a stable W/O emulsion system: PVTMS served as the oil-phase matrix, while an aqueous phase containing ammonia solution was gradually added dropwise during high-speed shear emulsification. Furthermore, catalyzed by ammonia, PVTMS underwent hydrolysis-condensation reactions and solidified into 3DM-PSSQ/H_2_O composite gel (Figure 1b) [41,42]. After high-temperature pyrolysis under argon atmosphere, the 3DM-PSSQ converted to the intermediate 3DM-SiO*_x_*/C accompanied by carbonization, while the emulsifier components simultaneously pyrolyzed to generate doped carbon. Finally, Ar/C_2_H_2_ gas mixture was introduced for CVD, forming a dense encapsulating carbon coating on the material surface to obtain the final product, 3DM-SiO*_x_*/C@C.

FT-IR spectroscopy was employed to monitor the progressive modification of functional groups during sample preparation (Figure 1c). VTMS and PVTMS exhibit characteristic peaks at 2850 and 1603 cm^−1^ corresponding to the stretching vibrations of -O-C-H and C=C bonds [43], respectively. The characteristic peak at 1603 cm^−1^ is similarly present in the 3DM-PSSQ, indicating that incompletely reacted VTMS monomers from radical polymerization participated in the subsequent sol–gel process. Significantly, 3DM-PSSQ exhibits characteristic absorption bands at 3400 and 1740 cm^−1^, corresponding to O-H and C=O stretching vibrations derived from the emulsifier [44]. After pyrolysis treatment, all the aforementioned characteristic peaks disappear, confirming the cleavage of Si-C bonds and complete pyrolysis/carbonization of the emulsifier. The final product 3DM-SiO*_x_*/C@C exhibits characteristic peaks at 1055, 809, and 467 cm^−1^, assigned to the asymmetric stretching, symmetric stretching, and bending vibrations of Si-O-Si bonds [45,46], respectively.

#### 3.1.2. Morphology and Pore Structure

**Figure 2:**
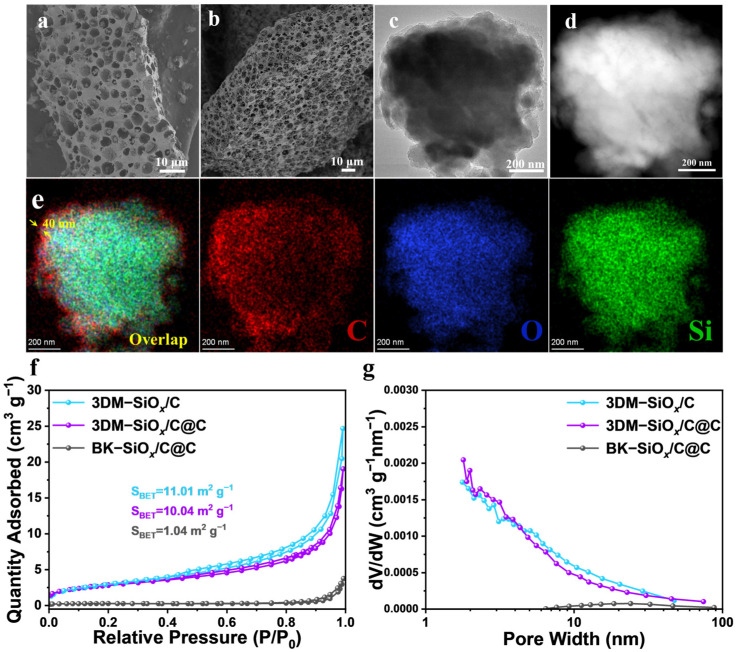
SEM images of (**a**) 3DM-SiO*_x_*/C@C and (**b**) 3DM-SiO*_x_*/C. (**c**) TEM, (**d**) HAADF-STEM, and (**e**) elemental mapping images of 3DM-SiO*_x_*/C@C. (**f**) N_2_ adsorption–desorption isotherms and (**g**) the corresponding pore-size distribution curves of 3DM-SiO*_x_*/C, 3DM-SiO*_x_*/C@C, and BK-SiO*_x_*/C@C.

The microstructure of the materials was characterized by SEM. After the sol–gel process and drying treatment, 3DM-PSSQ with a three-dimensional macroporous structure was formed (Figure S2a). Following pyrolysis and CVD treatment, the obtained 3DM-SiO*_x_*/C@C inherited the intact macroporous skeleton, while the PSSQ framework was in situ transformed into SiO*_x_*/C. The 3DM-SiO*_x_*/C@C possesses an abundance of macropores with diameters ranging from 2 to 5 μm (Figure 2a), and this macroporous architecture effectively buffers the volumetric expansion during cycling, while the enhanced specific surface area facilitates electron transport and lithium-ion diffusion. For comparison, 3DM-SiO*_x_*/C without a carbon coating (Figure 2b) and BK-SiO*_x_*/C@C without a macroporous structure (Figure S2b) were simultaneously prepared. Evidently, the 3DM-SiO*_x_*/C material also possesses abundant micron-scale macropores, whereas the BK-SiO*_x_*/C@C material shows no obvious porous structure. Elemental mapping tests confirm the homogeneous distribution of Si, C, and O elements in 3DM-SiO*_x_*/C@C (Figure S2c). The continuous C distribution demonstrates that a uniform carbon layer has been coated onto the 3DM-SiO*_x_*/C surface. Under identical testing conditions, the carbon signal intensity of 3DM-SiO*_x_*/C (Figure S2d) is significantly weaker than that of 3DM-SiO*_x_*/C@C. To comprehensively characterize the surface carbon coating, TEM was employed to analyze 3DM-SiO*_x_*/C@C and 3DM-SiO*_x_*/C. A carbon layer with a thickness of approximately 40 nm is coated on the surface of the 3DM-SiO*_x_*/C@C material (Figure 2c,d), and its elemental distribution maps clearly illustrate that the SiO*_x_* matrix is encapsulated within the carbon layer (Figure 2e). In contrast, no distinct carbon layer was observed in the 3DM-SiO*_x_*/C material (Figure S3a,b), and its homogeneous elemental distribution without localized C element accumulation (Figure S3c) further confirms its uncoated structural feature.

The specific surface areas and pore structures of three samples were analyzed via N_2_ adsorption–desorption isotherms, as depicted in Figure 2f, and the corresponding structural parameters are summarized in Table 1. When the relative pressure (P/P_0_) approaches 1, the isotherms of the 3DM-SiO*_x_*/C@C and 3DM-SiO*_x_*/C samples exhibit a sharp increase without forming a saturation plateau. Such reversible Type II isotherms typically appear in macroporous or nonporous materials [47]. Combined with the cross-sectional SEM images of the materials, this sharp increase is attributed to the presence of macropores within the 3DM architecture [48]. As shown in Table 1, the BET specific surface area of 3DM-SiO*_x_*/C@C (10.04 m^2^ g^−1^) exceeds that of BK-SiO*_x_*/C@C (1.04 m^2^ g^−1^) by nearly an order of magnitude, with 3DM-SiO*_x_*/C (11.01 m^2^ g^−1^) showing comparable enhancement, resulting from the enhanced porosity provided by their macroporous structures. The higher specific surface area increases active sites, establishes transport channels for rapid Li^+^ diffusion, and promotes accelerated lithiation/delithiation kinetics [49]. Both the 3DM-SiO*_x_*/C@C and 3DM-SiO*_x_*/C samples demonstrate micropores and mesopores within the 1~5 nm range (Figure 2g), attributed to volatile byproduct evolution during PSSQ pyrolysis. To quantitatively evaluate these fine pore structures, the mesopore and micropore volumes were calculated using the Barrett–Joyner–Halenda (BJH) and t-plot methods, respectively. For the 3DM-SiO*_x_*/C@C sample, the calculated micropore volume is extremely low (0.0003 cm^3^ g^−1^), and the mesopore volume is also highly limited (0.0302 cm^3^ g^−1^). The logically consistent co-existence of these micro/mesopores and the relatively low specific surface areas (10~11 m^2^ g^−1^) is fundamentally because their absolute pore volumes are negligible. The hierarchical structure is predominantly composed of the 3D macroporous framework. Compared to micropores, macropores contribute less to the specific surface area, while the dense CVD carbon coating further seals a portion of the initial fine surface pores.

#### 3.1.3. Composition and Carbon Structure

**Figure 3:**
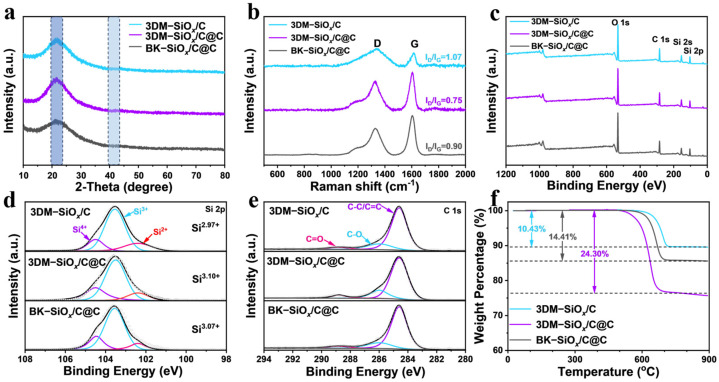
(**a**) XRD pattern, (**b**) Raman spectra, (**c**) XPS survey spectra, (**d**) Si 2p spectra, (**e**) C 1s spectra, and (**f**) TGA curves of 3DM-SiO*_x_*/C, 3DM-SiO*_x_*/C@C, and BK-SiO*_x_*/C@C.

XRD analysis was conducted to investigate the crystalline properties of SiO*_x_*/C composites. Figure 3a reveals a broad diffraction peak near 23° in all samples, corresponding to amorphous SiO*_x_* [45,50,51]. A weak broadened peak near 43° corresponds to the amorphous carbon component [52,53]. Thermal conversion of PSSQ to SiO*_x_* and carbon is evidenced under high-temperature conditions. Moreover, the graphitization degree of the samples was evaluated by Raman spectroscopy (Figure 3b). The characteristic peaks at 1608 and 1326 cm^−1^ correspond to the vibrational modes of graphitic carbon (G-band) and disordered carbon (D-band), respectively [54]. The *I*_D_/*I*_G_ ratios of CVD carbon-coated BK-SiO*_x_*/C@C and 3DM-SiO*_x_*/C@C samples are significantly lower than that of uncoated 3DM-SiO*_x_*/C, indicating a higher graphitization degree in the CVD-deposited carbon layer. Such structural features contribute to the enhanced electronic conductivity of the material [55].

Additionally, XPS was utilized to determine the surface chemistry of the comparative samples (3DM-SiO*_x_*/C, 3DM-SiO*_x_*/C@C, and BK-SiO*_x_*/C@C), as shown in Figure 3c–e. Survey spectra reveal that all three samples consist of C, O, and Si elements (Figure 3c). As shown in the high-resolution Si 2p spectra (Figure 3d), characteristic peaks are observed at 102.4, 103.5, and 104.5 eV for all three samples, corresponding to Si^2+^, Si^3+^, and Si^4+^ valence states [23,56,57], respectively. Integration of the peak areas enables the calculation of the average Si valence states as 2.97 (*x* = 1.485), 3.10 (*x* = 1.55), and 3.07 (*x* = 1.535) for 3DM-SiO*_x_*/C, 3DM-SiO*_x_*/C@C, and BK-SiO*_x_*/C@C, respectively. The calculated x values for the SiO*_x_* in all three materials consistently approach 1.5. This is attributed to the thermal pyrolysis of the PSSQ precursor (empirical formula (RSiO_1.5_)_n_), during which the Si-C bonds undergo cleavage while the Si-O-Si network is preserved [58]. The closely matched *x* values among these three comparative samples establish a reliable compositional baseline, ensuring that the subsequently observed differences in their electrochemical performances genuinely originate from the structural engineering (i.e., the 3DM framework and carbon coating) rather than stoichiometric discrepancies. The high-resolution C 1s spectrum (Figure 3e) displays common characteristic peaks at 284.6, 286.0, and 288.8 eV, assigned to C-C/C=C, C-O, and C=O bonds [59], respectively. These chemical bonds arise from the pyrolytic decomposition of vinyl groups and emulsifier, combined with carbon deposition via the CVD process.

The carbon contents of the samples were quantified by TGA. As illustrated in Figure 3f, all samples undergo substantial weight loss in the temperature range up to 500 °C, attributed primarily to the oxidation of carbon. Concurrently, the oxidation of low-valence silicon species into SiO_2_ induces a mass gain. To ensure objective quantification, this theoretical mass gain was accounted for based on the average *x* value (*x* ≈ 1.5) determined by the XPS analysis. Through mass balance calculations, the carbon contents in 3DM-SiO*_x_*/C, 3DM-SiO*_x_*/C@C, and BK-SiO*_x_*/C@C were determined to be 22.37 wt%, 34.39 wt%, and 26.28 wt%, respectively, and the mass of the CVD-deposited carbon was further deduced to be 0.1549 g per 1 g of the 3DM-SiOx/C@C composite (the calculation process is provided in Note S1). Compared with BK-SiO*_x_*/C@C, the elevated carbon content in the 3DM-SiO*_x_*/C@C material is primarily attributed to the increased surface area of the 3DM architecture, which provides more active sites for CVD carbon deposition. To provide a clear overview, the key compositional and structural parameters discussed above, including the carbon content, Si valence state, *x* value, and the *I*_D_/*I*_G_ ratio, are summarized in Table 2.

### 3.2. Electrochemical Performances

**Figure 4:**
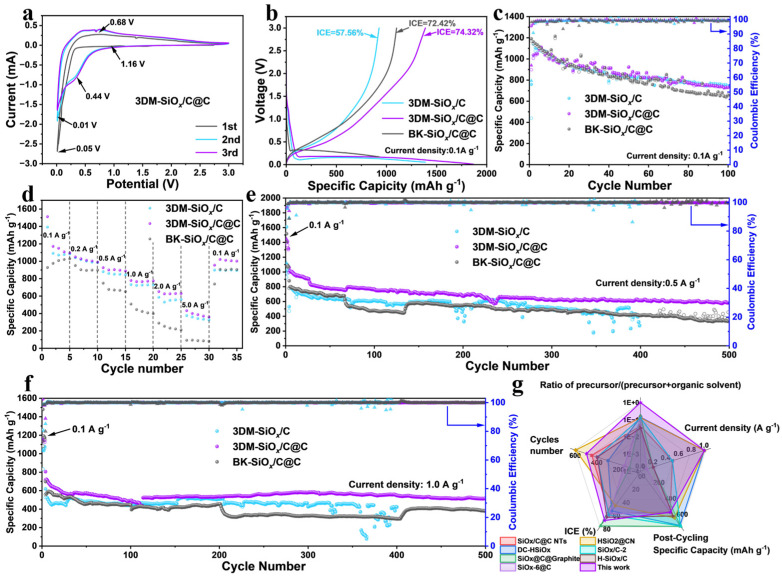
(**a**) CV curves of 3DM-SiO*_x_*/C@C, (**b**) primary charge–discharge curves at 0.1 A g^−1^, (**c**) the cycling performances at 0.1 A g^−1^ over 100 cycles, and (**d**) rate performances of the 3DM-SiO*_x_*/C@C, 3DM-SiO*_x_*/C, and BK-SiO*_x_*/C@C. The extended cycling stability of the 3DM-SiO*_x_*/C@C, 3DM-SiO*_x_*/C and BK-SiO*_x_*/C@C at current densities of (**e**) 0.5 A g^−1^ and (**f**) 1.0 A g^−1^. (**g**) Solvent consumption and electrochemical performance comparison with reported materials.

To evaluate the electrochemical reactions of 3DM-SiO*_x_*/C@C, 3DM-SiO*_x_*/C, and BK-SiO*_x_*/C@C materials during charge/discharge processes, cyclic voltammetry (CV) tests were performed on these electrodes (Figure 4a and Figure S4). In the first three cycles of the 3DM-SiO*_x_*/C@C electrode, the cathodic peak observed at 1.16 V is attributed to electrolyte decomposition forming an SEI film. This peak vanishes in following cycles, evidencing the establishment of a stable SEI during the first cycle, which reduces excessive Li^+^ consumption in subsequent cycles. The peak at 0.05 V corresponds to the lithiation of SiO*_x_*, involving reactions generating Li_2_O, lithium silicate salts, and Si-Li alloy (Equations (1)–(3)) [60,61]. In subsequent cycles, this peak splits into two peaks at 0.01 V and 0.44 V, corresponding to reversible alloying reactions. The anodic peak at 0.68 V is ascribed to the dealloying reaction of Li*_x_*Si. In the following two cycles, the CV curves of the 3DM-SiOx/C@C electrode overlap well, demonstrating significantly better overlap than those of the 3DM-SiO*_x_*/C and BK-SiO*_x_*/C@C electrodes (Figure S4). This indicates the superior reversibility of the 3DM-SiO*_x_*/C@C material compared to the 3DM-SiO*_x_*/C and BK-SiO*_x_*/C@C materials.

(1) $$SiO_{x}+2xLi^{+}+2xe^{-}\to xLi_{2}O+Si$$

(2) $${SiO}_{x}+{xLi}^{+}+{xe}^{-}\to \left(x/4\right){Li}_{4}{SiO}_{4}+\left(1-x/4\right)Si$$

(3) $$Si+{xLi}^{+}+{xe}^{-}↔{Li}_{x}Si$$

To compare the electrochemical behavior of the three materials under low current density, initial charge–discharge and short-cycle tests were conducted at 0.1 A g^−1^ (Figure 4b,c). The initial cycling performance of 3DM-SiO*_x_*/C exhibits a discharge capacity of 1608.8 mAh g^−1^ versus a charge capacity of 926 mAh g^−1^, resulting in 57.56% ICE. This reversible capacity is notably lower than the theoretical limit of pure SiO*_x_* (~2000 mAh g^−1^), which is intrinsically constrained by the mass dilution of the initial carbon component and further exacerbated by the incomplete utilization of active SiO*_x_* due to poor electrical conductivity [62]. The low ICE originates from the absence of a carbon coating on 3DM-SiO*_x_*/C and its increased specific surface area. This enlarged interfacial contact with electrolyte accelerates parasitic side reactions, causing irreversible active lithium loss—a critical limitation for commercial lithium-ion batteries. After carbon coating, the 3DM-SiO*_x_*/C@C material delivers an initial discharge capacity of 1866.7 mAh g^−1^ with an ICE of 74.32%, despite a higher carbon content that theoretically dilutes the overall capacity. This is ascribed to the robust conductive network constructed by CVD carbon, which electrically activates the isolated inner SiO*_x_*, thereby maximizing the active material utilization. The carbon coating acts as a robust physical barrier, preventing direct contact between the inner SiOx components and the electrolyte. Consequently, a stable and uniform SEI layer primarily forms on the outer carbon surface rather than on the highly reactive SiO*_x_*. This protective carbon encapsulation effectively suppresses the irreversible parasitic side reactions occurring on SiO*_x_* during the initial lithiation process, thereby preventing the excessive consumption of active lithium ions and significantly improving the ICE [63,64]. Subsequently, the CE climbs to 96.99% by the second cycle and maintains stability exceeding 99% beyond ten cycles. Notably, 3DM-SiO*_x_*/C@C exhibits a higher ICE compared to the non-porous BK-SiO*_x_*/C@C (72.42%), despite its significantly larger specific surface area. For the non-porous BK-SiO*_x_*/C@C, the lack of buffer space generates massive internal stress during the initial lithiation, which can induce cracks or even structural rupture in the protective carbon coating. This early compromise of the physical barrier inevitably leads to massive exposure of highly reactive fresh SiO*_x_* surfaces to the electrolyte, accelerating irreversible side reactions and excessive initial SEI formation. In contrast, the robust 3DM architecture effectively preserves the integrity of the carbon shell, thereby preventing excessive active lithium loss and boosting the ICE.

Rate capability measurements of the three electrodes were performed at stepped current densities (0.1, 0.2, 0.5, 1.0, 2.0, and 5.0 A g^−1^) to assess how the 3DM structure and carbon coating influence material capacity and fast-charging performance. As shown in Figure 4d, as the current density increases, the 3DM-SiO*_x_*/C@C electrode exhibits specific capacities of 1094.9, 990.1, 888.4, 774.4, 628.9, and 358.3 mAh g^−1^ after five cycles at each respective rate. This performance is significantly superior to that of the BK-SiO*_x_*/C@C and also surpasses that of the uncoated 3DM-SiO*_x_*/C. Upon reverting to 0.1 A g^−1^, the specific capacity restored to 1001.5 mAh g^−1^, demonstrating a 91.5% capacity recovery ratio. This value once again surpasses those of the other two materials, fully demonstrating the outstanding electrochemical reversibility of 3DM-SiO*_x_*/C@C.

To assess the long-term cycling stability of the three materials, extended cycling tests were performed on 3DM-SiO*_x_*/C@C, 3DM-SiO*_x_*/C, and BK-SiO*_x_*/C@C electrodes at 0.5 A g^−1^ (Figure 4e). The 3DM-SiO*_x_*/C@C anode displays a discharge-specific capacity of 1057.9 mAh g^−1^ in the fourth cycle (which serves as the baseline capacity, being the first cycle at the target current density after three initial activation cycles at 0.1 A g^−1^), and maintains 584.6 mAh g^−1^ after 500 charge/discharge cycles at 0.5 A g^−1^, corresponding to a capacity retention of 55.3%. In contrast, the BK-SiO*_x_*/C@C anode exhibits only 39.2% capacity retention after 500 cycles, with frequent Coulombic efficiency instability observed after 400 cycles. The absence of a macroporous structure in BK-SiO*_x_*/C@C leads to gradual pulverization during cycling due to repeated volume changes, while additional SEI layer accumulation consumes substantial active lithium ions, resulting in capacity decay and unstable Coulombic efficiency in later cycles. Moreover, under 1.0 A g^−1^ cycling conditions (Figure 4f), the 3DM-SiO*_x_*/C@C electrode maintains a 515.9 mAh g^−1^ discharge capacity after 500 cycles, demonstrating 71.4% capacity retention (calculated based on the 4th cycle capacity of 722 mAh g^−1^, following the same initial activation protocol) with a minimal cycle-by-cycle decay of 0.057%, emphasizing the critical role of controlled nanostructural engineering.

To comprehensively demonstrate the all-round performance profile of the 3DM-SiO*_x_*/C@C anode, a radar chart (Figure 4g) is employed to systematically compare its performance across five key metrics—ICE, post-cycling specific capacity, current density, cycle number, and the precursor-to-solvent mass ratio—with previously reported SiO*_x_*-based anodes [53,61,65,66,67,68,69]. Our material exhibits a well-balanced electrochemical performance while demonstrating distinct advantages in terms of green synthesis. A detailed evaluation of this green strategy, including comparisons of solvent types/amounts, template removal parameters, and estimated waste generation, is summarized in Table S3. As evidenced by the precursor-to-solvent mass ratio in Figure 4g and the data in Table S3, the 3DM-SiO*_x_*/C@C synthesis uniquely employs no organic solvents or washing steps throughout the entire process, achieving a ratio of 1. In contrast, all other referenced materials require large quantities of organic solvents, with precursor mass ratios ranging merely between 0.0044 and 0.17. Furthermore, compared with reported macroporous structural materials prepared via hard-templating methods [23,32,33,34,35,36], this method eliminates template synthesis/removal procedures, representing a truly green and facile synthetic approach.

### 3.3. Electrochemical Kinetic Analysis

**Figure 5:**
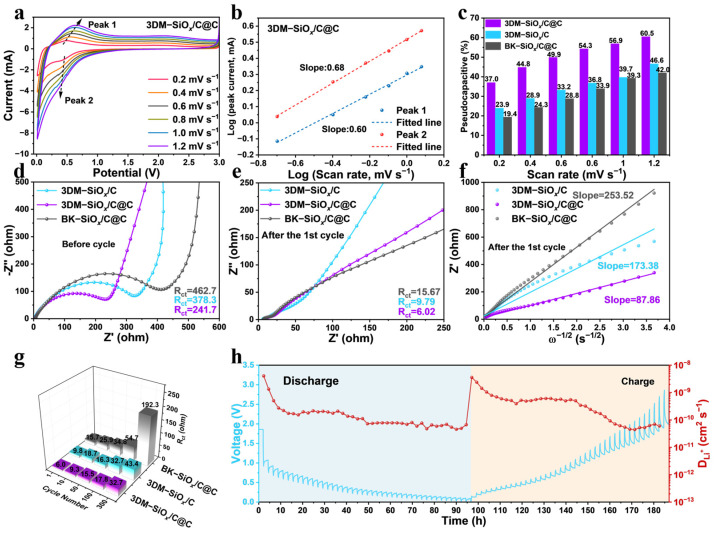
(**a**) CV profiles and (**b**) log *i* vs. log *v* plots at different scan rates of 3DM-SiO*_x_*/C@C. (**c**) Percentage of capacitive-controlled capacities, (**d**) Nyquist curves before cycling, (**e**) Nyquist plots after the 1st cycling, (**f**) the relation of Z′-ω^−1/2^ plots at the low frequency, (**g**) variation in the R_ct_-value after 300 cycles of high rate charge/discharge of 3DM-SiO*_x_*/C@C, 3DM-SiO*_x_*/C and BK-SiO*_x_*/C@C. (**h**) GITT curves of 3DM-SiO*_x_*/C@C.

Cyclic voltammetry measurements were performed at multiple scan rates to elucidate the origin of the superior stability in 3DM-SiO*_x_*/C@C anode (Figure 5a). Within the scan rate range of 0.2 to 1.2 mV s^−1^, all CV curves exhibit similar trends (Figure S5a,c). The current intensity (*i*) shows a positive correlation with the scan rate (*v*) (Figure S5b,d). The specific relationship is shown in Equations (4) and (5) [70]:

(4) $$i=av^{b}$$

(5) $$log\left(i\right)=blog\left(v\right)+log\left(a\right)$$

where *a* and *b* are adjustable constants, the *b*-value can be obtained by plotting log(*i*) versus log(*v*) and calculating its slope.

When the electrochemical reaction is dominated by diffusion behavior, the *b*-value equals 0.5, whereas when the charge storage process is purely pseudocapacitive, the *b*-value reaches 1.0 [71]. The calculated *b*-values for the cathodic peak and anodic peak of 3DM-SiO*_x_*/C@C are 0.68 and 0.60, respectively (Figure 5b), indicating that lithium-ion storage is jointly dominated by diffusion behavior and the pseudocapacitive effect. Current research indicates that the pseudocapacitive effect plays a beneficial role in lithium-ion storage at high current densities [72]. Moreover, both 3DM-SiO*_x_*/C@C and 3DM-SiO*_x_*/C exhibit higher b-values relative to those of the BK-SiO*_x_*/C@C, demonstrating that the macroporous structure can effectively increase the proportion of the pseudocapacitive effect in lithium-ion storage. The fractional contributions of pseudocapacitive and diffusion-controlled processes to total capacitance are precisely determined through Equations (6) and (7) [73]:

(6) $$i\left(v\right)=k_{1}v+k_{2}v^{1/2}$$

(7) $$i\left(v\right)/v^{1/2}=k_{1}v^{1/2}+k_{2}$$

Within the scan rate range of 0.2 to 1.2 mV s^−1^, the contribution ratios of the pseudocapacitive effect for 3DM-SiO*_x_*/C@C, 3DM-SiO*_x_*/C, and BK-SiO*_x_*/C@C electrodes were calculated (Figure 5c). The results demonstrate that as the scan rate increases, the contribution ratio of the pseudocapacitive effect rises accordingly, with the percentages of pseudocapacitive contribution in 3DM-SiO*_x_*/C@C and 3DM-SiO*_x_*/C electrodes consistently higher than that of BK-SiO*_x_*/C@C. Furthermore, at a scan rate of 1.2 mV s^−1^, the pseudocapacitive contributions of 3DM-SiO*_x_*/C@C (60.5%) and 3DM-SiO*_x_*/C (46.6%) both exceed that of the BK-SiO*_x_*/C@C electrode (Figure S6), indicating that the macroporous structure with a large specific surface area provides abundant active sites for rapid lithium-ion insertion/extraction, thereby accelerating electrochemical reaction kinetics [74].

To probe electrode kinetics further, electrochemical impedance spectroscopy (EIS) was conducted across multiple cycle states for all three electrodes (Figure 5d–f). The EIS spectra consist of a low-frequency oblique line characteristic of Warburg impedance (*Z_w_*) and a high-frequency semicircle reflecting charge transfer resistance (*R_ct_*), with *R_ct_* values quantified using the equivalent circuit in Figure S7 [75]. Before cycling, the 3DM-SiO*_x_*/C@C electrode exhibits a significantly lower *R*_ct_ (241.7 Ω) than both 3DM-SiO*_x_*/C (378.3 Ω) and BK-SiO*_x_*/C@C (462.7 Ω) (Figure 5d), indicating that electron transport is enhanced due to the carbon coating and enlarged surface area from the porous structure. After the first charge/discharge cycle, all materials show substantial *R*_ct_ reduction (Figure 5e), which is primarily attributed to enhanced surface reactivity and facilitated charge transfer resulting from the initial SEI formation [76]. Furthermore, the Warburg coefficient (σ) was derived from linear fitting of Z′-ω^−1/2^ plots (ω = 2πf), which inversely correlates with the apparent lithium-ion diffusion rate (Figure 5f). The fitted σ values for 3DM-SiO*_x_*/C@C, 3DM-SiO*_x_*/C, and BK-SiO*_x_*/C@C are 87.86, 173.38, and 253.52, respectively, demonstrating the fastest Li^+^ diffusion kinetics in 3DM-SiO*_x_*/C@C [77]. The *R*_ct_ values of electrodes after different cycling stages were measured to evaluate electrode-electrolyte stability during long-term cycling, as shown in Figure 5g and Figure S8. During 300 charge/discharge cycles, the 3DM-SiO*_x_*/C@C electrode exhibits exceptional stability in *R*_ct_ values, with the value increasing from an initial 6.0 Ω to 32.7 Ω, demonstrating the stability of its surface SEI layer. In contrast, the absence of a protective carbon coating results in a greater *R*_ct_ increase (from 9.8 Ω to 43.4 Ω) for 3DM-SiO*_x_*/C than for 3DM-SiO*_x_*/C@C. Nevertheless, its *R*_ct_ values remain significantly lower than those of the BK-SiO*_x_*/C@C electrode (from 15.7 Ω to 192.3 Ω). This phenomenon is attributed to excessive SEI accumulation on BK-SiO*_x_*/C@C during cycling, resulting from insufficient internal space to accommodate volume changes, which impedes interfacial electron/Li^+^ exchange, thereby highlighting the structural merits of the three-dimensional macroporous architecture. To quantitatively evaluate the lithium-ion diffusion rate, the galvanostatic intermittent titration technique (GITT) was employed to determine the lithium-ion diffusion coefficient of the electrodes. The specific values were obtained using the simplified formula, Equation (8) [78,79]:

(8) $$D_{{Li}^{+}}=\frac{4}{\pi \tau }{\left(\frac{m_{B}V_{m}}{M_{B}S}\right)}^{2}{\left(\frac{∆E_{S}}{∆E_{\tau }}\right)}^{2}$$

where τ, $m_{B}$, $M_{B}$, $V_{m}$ and *S* represent the pulse duration, mass of the active material, molar mass, molar volume, and active surface area of the electrode, respectively; $∆E_{s}$ and $∆E_{\tau }$ denote the potential changes caused by relaxation and galvanostatic charging/discharging, respectively. As shown in Figure 5h and Figure S9, the calculated average $D_{{Li}^{+}}$ values for 3DM-SiO*_x_*/C@C and 3DM-SiO*_x_*/C samples are 3.85 × 10^−10^ cm^2^ s^−1^ and 3.43 × 10^−10^ cm^2^ s^−1^, respectively, both higher than that of the BK-SiO*_x_*/C@C sample (2.10 × 10^−10^ cm^2^ s^−1^). This indicates that the presence of a 3D macroporous architecture facilitates highly efficient Li^+^ transport, enhancing the lithiation/delithiation kinetics and providing robust kinetic support for higher electrode mass loading.

**Figure 6:**
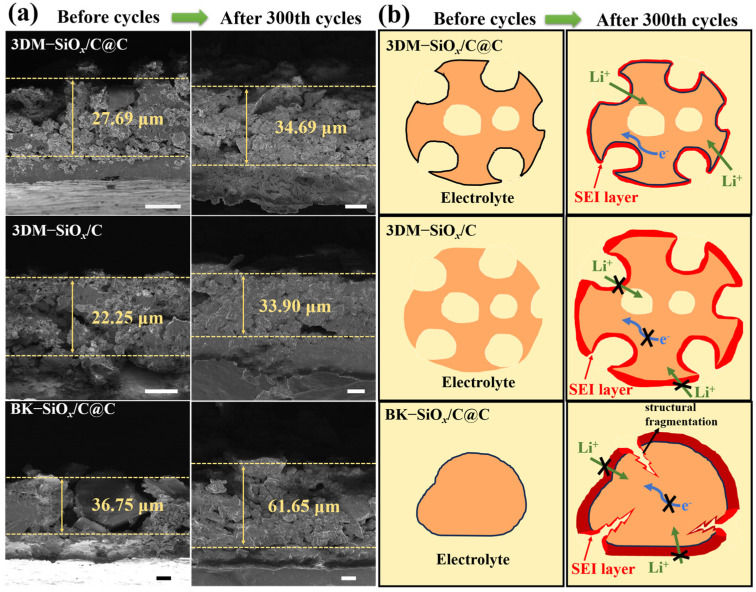
(**a**) Cross-sectional SEM images and (**b**) the evolution diagram of 3DM-SiO*_x_*/C@C, 3DM-SiO*_x_*/C, and BK-SiO*_x_*/C@C before (**left**) and after (**right**) 300 cycles. All scale bars are 10 μm.

To investigate morphological and dimensional changes in electrodes during charge/discharge cycles, SEM characterization of three electrode materials after 300 cycles at 1.0 A g^−1^ was performed, revealing that BK-SiO*_x_*/C@C exhibits substantial expansion (67.76%, Figure 6a) with its surface covered by a thick SEI layer featuring cracks (Figure S10a), which is attributable to volumetric variation in SiO*_x_* causing particle extrusion, structural fragmentation, fresh surface exposure, and repetitive SEI formation (Figure 6b). While the porous structure of 3DM-SiO*_x_*/C partially suppressed expansion, the absence of carbon coating led to continuous active surface exposure during lithiation, resulting in SEI accumulation, crack formation (Figure 6a and Figure S10b), and severely impeded Li^+^/electron transport due to 52.3% expansion. In contrast, 3DM-SiO*_x_*/C@C shows only 25.28% expansion (Figure 6a) without cracks or thick SEI (Figure S10c), confirming structural stability (Figure 6b) due to the following reasons: (1) 3D macropores buffering volume changes while shortening Li^+^ paths; (2) carbon coating enabling stable SEI formation without accumulation; (3) enhanced conductivity/diffusion. XPS analysis of cycled 3DM-SiO*_x_*/C@C (Figure S11) identifies LiF, Li_2_O, and lithium silicates as primary SEI components [31].

## 4. Conclusions

In summary, a 3DM-SiO*_x_*/C@C nanocomposite featuring three-dimensional macropores and carbon coating was developed through water-in-oil emulsion templating combined with sol–gel processing and chemical vapor deposition. Compared with conventional hard-templating methods for constructing 3D macroporous structures, this approach eliminates complex steps involving template fabrication and removal while requiring no organic solvents throughout the synthesis, rendering the process both simple and environmentally benign. The macropore architecture effectively accommodates volumetric variations during cycling, while the carbon coating promotes the formation of a stable SEI layer that mitigates excessive active lithium consumption. These advantages collectively enable the 3DM-SiO*_x_*/C@C anode to deliver an initial Coulombic efficiency of 74.32% and maintain a reversible capacity of 515.9 mAh g^−1^ after 500 cycles at 1.0 A g^−1^. Additionally, the electrode exhibits minimal thickness variation (~25%) before and after cycling. While the current performance is evaluated at a baseline mass loading of about 1.0 mg cm^−2^ to accurately unveil its intrinsic electrochemical properties and structural advantages, this 3D macroporous architecture inherently possesses the scalable potential to support higher mass loadings by facilitating efficient electron and ion transport within the electrode. However, we acknowledge that achieving commercial-level high mass loadings presents practical scalability limitations and distinct electrode engineering challenges, such as potential electrolyte flooding within the macroporous voids and the resulting excessive electrolyte consumption. Furthermore, regarding cost analysis, while our organic solvent-free and template-free approach significantly minimizes raw material and waste treatment expenses, further optimization of the CVD process is required to reduce manufacturing costs at scale. Addressing these challenges and maintaining the current excellent performance in thicker electrodes will be a critical focus of our future work. This work facilitates the transition of 3D macroporous materials from laboratory prototypes toward industrial applications, showing promise for commercialization.

## Figures and Tables

**Table 1:** Porous structure parameters of 3DM-SiO*_x_*/C, 3DM-SiO*_x_*/C@C, and BK-SiO*_x_*/C@C.

Sample	BET Surface Area (m^2^ g^−1^)	Pore Volume (cm^3^ g^−1^)	Average Pore Size (nm)
3DM-SiO*_x_*/C	11.01	0.0383	13.90
3DM-SiO*_x_*/C@C	10.04	0.0302	11.58
BK-SiO*_x_*/C@C	1.04	0.0058	52.34

**Table 2:** Compositional and structural parameters of 3DM-SiO*_x_*/C, 3DM-SiO*_x_*/C@C, and BK-SiO*_x_*/C@C.

Sample	Carbon Content (wt%)	Si Valence State	*x* Value	*I*_D_/*I*_G_ Ratio
3DM-SiO*_x_*/C	22.37	2.97	1.485	1.07
3DM-SiO*_x_*/C@C	34.39	3.10	1.550	0.75
BK-SiO*_x_*/C@C	26.28	3.07	1.535	0.90

## Data Availability

The raw data supporting the conclusions of this article will be made available by the authors on request.

## Supplementary Materials

The following supporting information can be downloaded at: https://www.mdpi.com/article/10.3390/polym18111398/s1, Figure S1: GPC curve of PVTMS; Figure S2: SEM images of (a) 3DM-PSSQ and (b) BK-SiO*_x_*/C@C. Elemental mapping (Contains C, O, and Si) of (c) 3DM-SiO*_x_*/C@C and (d) 3DM-SiO*_x_*/C; Figure S3: (a) TEM, (b) HAADF-STEM, and (c) elemental mapping images of 3DM-SiO*_x_*/C; Figure S4: CV curves of (a) 3DM-SiO*_x_*/C and (b) BK-SiO*_x_*/C@C; Figure S5: CV profiles and log *i* vs. log *v* plots at different scan rates of (a,b) 3DM-SiO*_x_*/C and (c,d) BK-SiO*_x_*/C@C; Figure S6: The capacity contribution at 1.2 mV s^−1^ of (a) 3DM-SiO*_x_*/C@C, (b) 3DM-SiO*_x_*/C, and (c) BK-SiO*_x_*/C@C; Figure S7: The equivalent circuit for data fitting; Figure S8: Nyquist plots after different cycles of (a) 3DM-SiO*_x_*/C@C, (b) 3DM-SiO*_x_*/C, and (c) BK-SiO*_x_*/C@C; Figure S9: GITT curves of (a) 3DM-SiO*_x_*/C and (b) BK-SiO*_x_*/C@C; Figure S10: SEI images of the electrode surface after 300 cycles: (a) BK-SiO*_x_*/C@C, (b) 3DM-SiO*_x_*/C, and (c) 3DM-SiO*_x_*/C@C; Figure S11: XPS spectra of the (a) Si 2p, (b) F 1s, and (c) Li 1s in 3DM-SiO*_x_*/C@C electrode after cycles; Note S1: Calculation of the CVD-deposited carbon mass; Table S1: Molecular weight averages of PVTMS; Table S2: Comparison of various templating strategies for the fabrication of porous silicon-based anodes; Table S3: Comparison of different synthetic strategies, solvent consumption, and scalability for typical SiO*_x_*-based anode materials.
