# Supplementary material for: Organic Solvent-Free and Emulsion Self-Templating Synthesis of 3D Macroporous SiOx/C@C for Durable Lithium-Ion Battery Anodes
Source: Polymers (Basel). 2026 Jun 4;18(11):1398. doi: 10.3390/polym18111398 (PMC13259472; doi:10.3390/polym18111398)
Supplement: Supplementary file 1 [file polymers-18-01398-s001.zip › polymers-4307179-supplementary.pdf]

Supporting information

Organic Solvent-Free and Emulsion Self-Templating Synthesis of 3D Macroporous SiO_x/C@C for Durable Lithium-Ion Battery Anodes

Jianing Zong[1, a], *Kaize Si*[1, a], *Jingjing Li*[a], *Xiaomei Wang*[a,*] and *Xu Zhang*[a, *]

[a] Hebei key Laboratory of Functional Polymers, School of Chemical Engineering and Technology, Hebei University of Technology, Tianjin 300130, P.R. China

*Corresponding author: E-mail: xmwang@hebut.edu.cn and xuzhang@hebut.edu.cn

[1]These two authors equally contributed to this work.

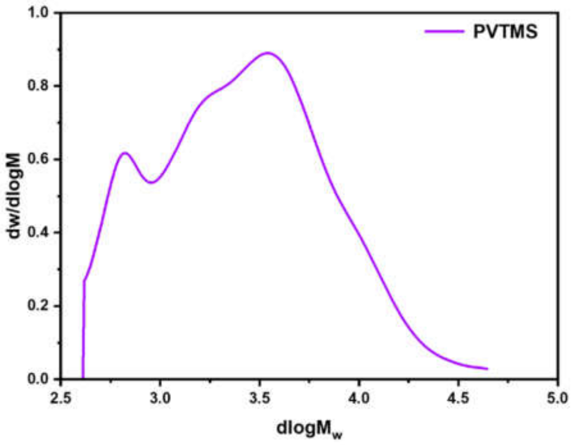

Fig. S1. GPC curve of PVTMS.

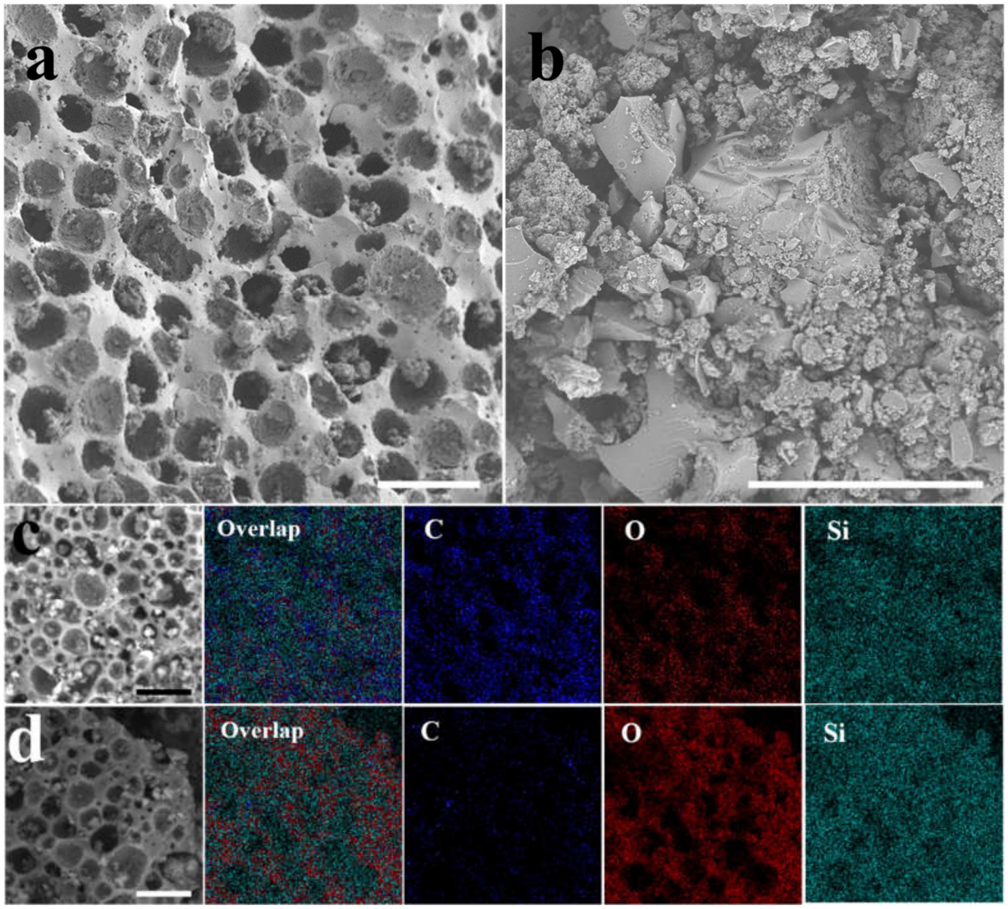

Fig. S2. SEM images of (a) 3DM-PSSQ and (b) BK-SiO_x/C@C. Elemental mapping (Contains C, O, and Si) of (c) 3DM-SiOx/C@C and (d) 3DM-SiOx/C. All the scale bars, 10 μm.

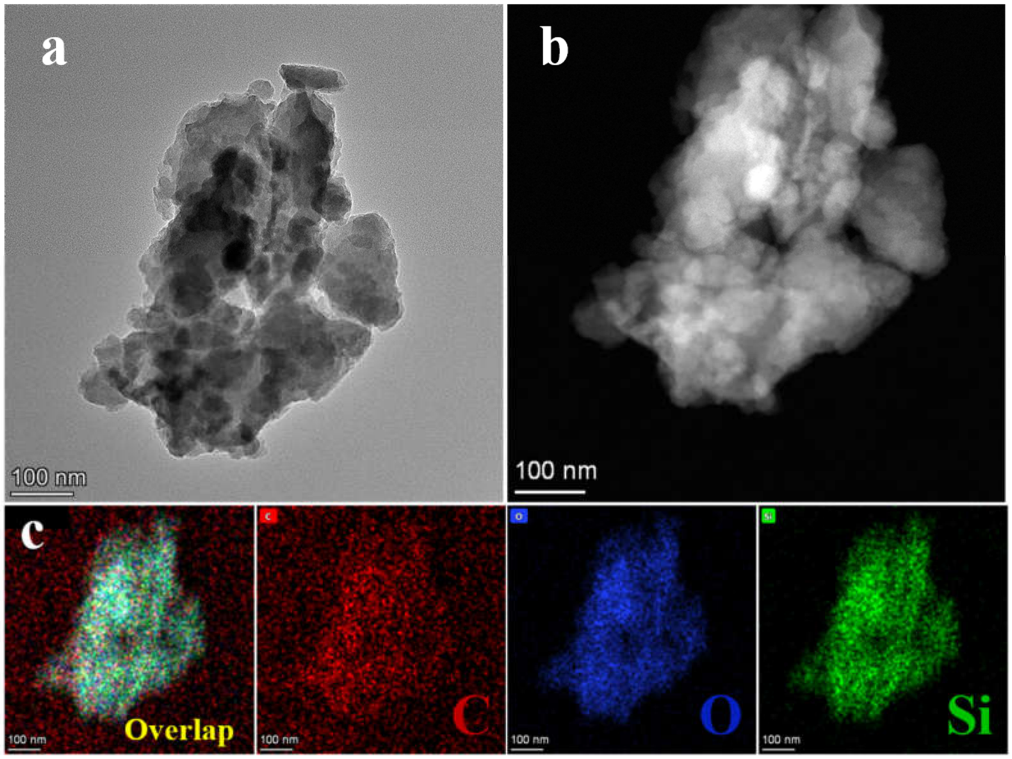

Fig. S3. (a) TEM, (b) HAADF-STEM, and (c) elemental mapping images of 3DM-SiO_x/C.

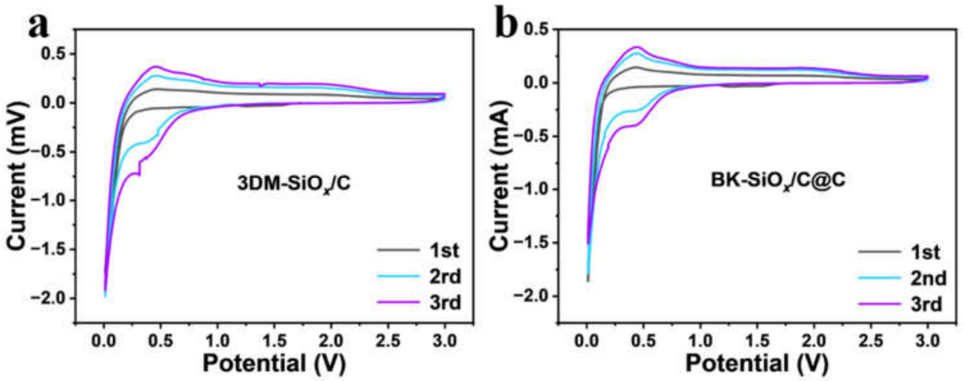

Fig. S4. CV curves of (a) 3DM-SiO$_x$/C and (b) BK-SiO$_x$/C@C.

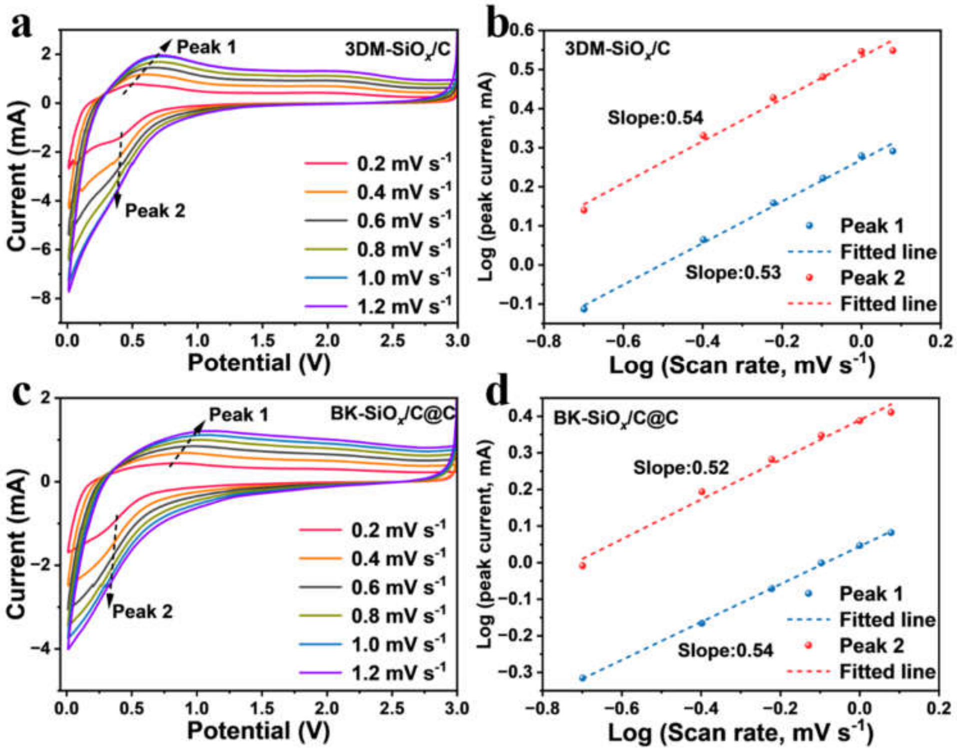

Fig. S5. CV profiles and log i vs. log v plots at different scan rates of (a,b) 3DM-SiO_x/C and (c,d) BK-SiO_x/C@C

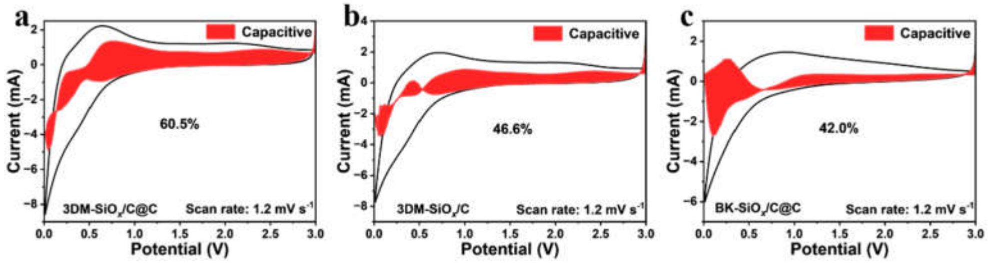

Fig. S6. The capacity contribution at 1.2 mV s^{-1} of (a) 3DM-SiO_x/C@C, (b) 3DM-SiO_x/C, and (c) BK-SiO_x/C@C.

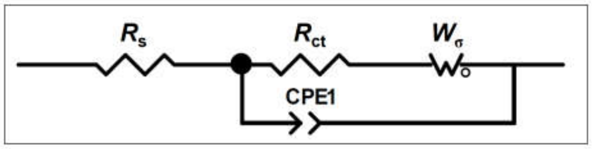

Fig. S7. The equivalent circuit for data fitting.

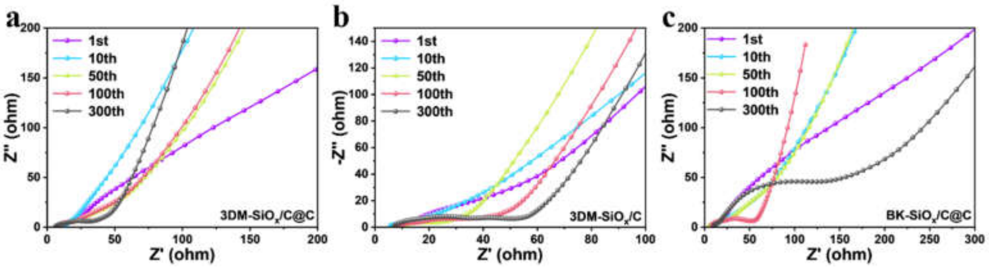

Fig. S8. Nyquist plots after different cycles of (a) 3DM-SiO$_x$/C@C, (b) 3DM-SiO$_x$/C, and (c) BK-SiO$_x$/C@C.

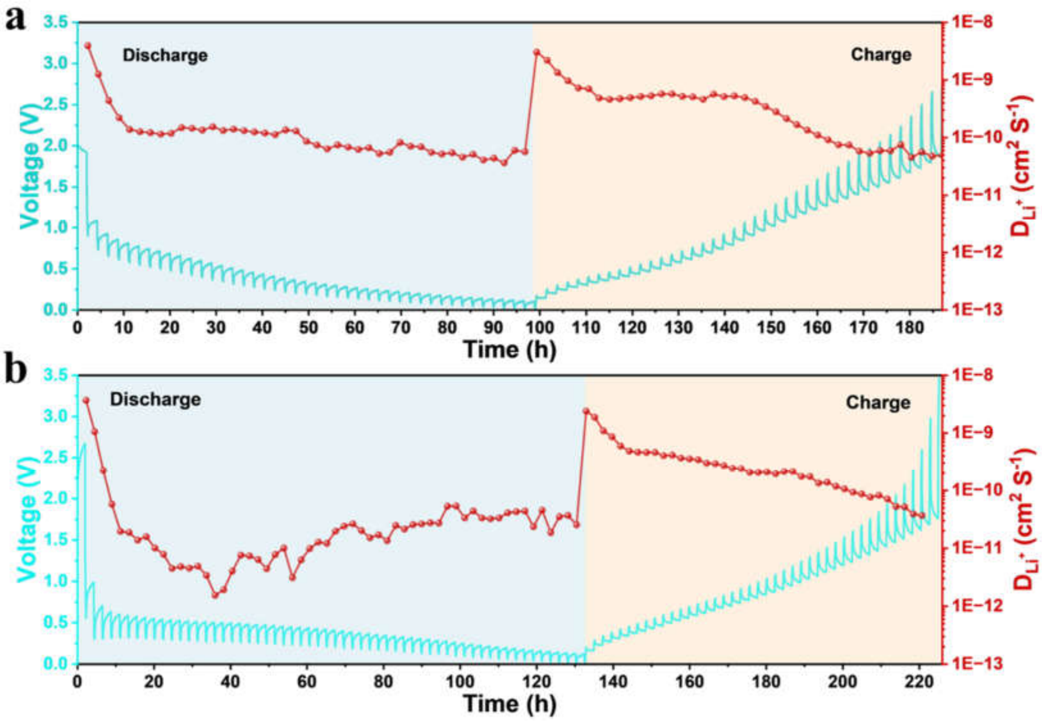

Fig. S9. GITT curves of (a) 3DM-SiO_x/C and (b) BK-SiO_x/C@C.

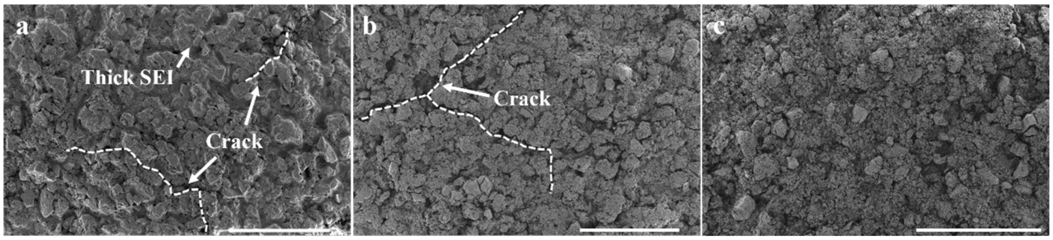

Fig. S10. SEI images of the electrode surface after 300 cycles: (a) BK-SiO_x/C@C, (b) 3DM-SiO_x/C, and (c) 3DM-SiO_x/C@C. All the scale bars are 150 μm.

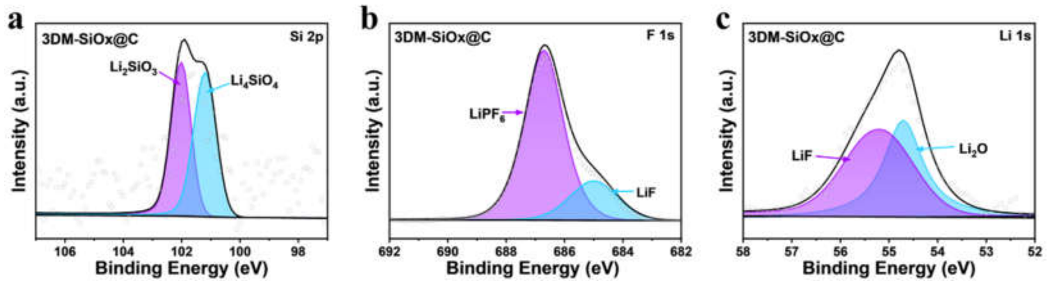

Fig. S11. XPS spectra of the (a) Si 2p, (b) F 1s, and (c) Li 1s in 3DM-SiO_x/C@C electrode after cycles.

Note S1: Calculation of the CVD-deposited carbon mass

The amount of CVD-deposited carbon in the final 3DM-SiO_x/C@C composite was determined by a mass-balance method based on the invariant mass of the SiOx matrix before and after the CVD process. During the TGA testing in air, the SiO_x matrix ($x \approx 1.5$, determined by XPS) undergoes complete oxidation to form SiO_2. Based on the molar mass change from $SiO_{1.5}$ to SiO_2, the initial SiO_x matrix mass can be deduced from the residual SiO_2 mass.

(1) For the intermediate 3DM-SiO_x/C material: The TGA residual SiO_2 content is 89.57 wt%. Thus, the intrinsic SiO_x matrix content is calculated to be 77.63 wt%, yielding an intrinsic pyrolytic carbon content of 22.37 wt%. The mass ratio of intrinsic pyrolytic carbon to SiO_x is 22.37 / 77.63 = 0.2882.

(2) For the final 3DM-SiO_x/C@C material: The TGA residual SiO_2 content is 75.70 wt%, corresponding to a SiO_x matrix content of 0.6561 g and a total carbon content of 0.3439 g per 1 g of the final product. By applying the intrinsic mass ratio (0.2882) to the 0.6561 g of SiO_x in the final product, the mass of the intrinsic pyrolytic carbon in 1 g of the final composite is calculated as: 0.6561 g × 0.2882 = 0.1890 g.

Consequently, the mass of the newly deposited CVD carbon is the difference between the total carbon and the intrinsic pyrolytic carbon: 0.3439 g − 0.1890 g = 0.1549 g.

Therefore, the exact mass of CVD-deposited carbon per 1 g of the final 3DM-SiO_x/C@C composite is 0.1549 g (corresponding to an actual mass fraction of 15.49 wt% for the CVD carbon in the final product).

Tab. S1. Molecular weight averages of PVTMS.

Peak	M_ρ (g/mol)	M_n (g/mol)	M_w (g/mol)	M_z (g/mol)	M_{z+1} (g/mol)	M_v (g/mol)
Peak 1	3419	1698	4210	9929	18058	3654

Tab. S2. Comparison of various templating strategies for the fabrication of porous silicon-based anodes

**Material**	**Templating Strategy**	**Template Removal**	**Solvent Usage**	**Scalability**
P-SiO[1]	Hard (Si)	NaOH etching	Ethanol	Poor
NSR-2[2]	Hard (Si)	NaOH etching	Acetone	Poor
PSi-9[3]	Hard (Al)	HCl etching	Ethanol	Poor
3DOM-SC@C[4]	Hard (CLPS)	Pyrolysis	Ethanol	Moderate
3D-Si@SiO_x/C[5]	Hard (KIT-6)	Pyrolysis/HCl etching	Butanol/Ethanol	Very Poor
C-nSi/SiO_x@Cy[6]	Hard (Al)	HCl etching	Ethanol	Poor
Porous SiO_x[7]	Soft (Soybean Oil)	Pyrolysis	HCl Solution	Moderate
SiO_x@NC-2[8]	Soft (CTAB Micelles)	Ethanol washing	Ethanol	Moderate
This work	Self-templating emulsion	Freeze-drying	Water	High

Tab. S3. Comparison of different synthetic strategies, solvent consumption, and scalability for typical SiO_x-based anode materials.

Material	Synthesis Strategy	Organic Solvent & Amount	Template Removal	Estimated Waste Generation	Scalability
SiO_x/C-2[9]	Non-template	Ethanol (~8 mL g^{-1})	N/A	Mild organic waste liquid; Exhaust	Moderate
SiO_x/C@C NTs[10]	Hard-template	Cyclohexane (~18 mL g^{-1}), Dichloromethane (~39 mL g^{-1}), Ethanol (~150 mL g^{-1})	Thermal degradation (900 °C 3h)	Massive toxic waste liquid (Dichloromethane); Toxic exhaust	Poor
DC-HSiO_x[11]	Soft-template	Ethanol (~30 mL g^{-1})	Water soaking (Room temperature 3 h)	Massive organic waste liquid; Exhaust	Moderate
SiO_x@C@Graphite[12]	Non-template	Ethanol (~5 mL g^{-1})	N/A	Mild organic waste liquid; Exhaust	Moderate
SiO_x-6@C[13]	Hard-template	Ethanol (~120 mL g^{-1})	Thermal degradation (900 °C 3h)	Massive acidic waste liquid (H_2SO_4); Toxic exhaust; Exhaust	Poor
HSiO_2@CN[14]	Soft-template	Ethanol (~85 mL g^{-1})	Water soaking	Massive organic waste liquid; Exhaust	Moderate
H-SiO_x/C[15]	Soft-template	Ethanol (~40 mL g^{-1}), DMF (~25 mL g^{-1})	Water soaking (80 °C 10h)	Massive toxic waste liquid (DMF); Exhaust	Poor
This work	emulsion self-template	None	Freeze-Drying (-50 °C 24h)	Exhaust	High

Reference

[1] B.-C. Yu, Y. Hwa, J.-H. Kim and H.-J. Sohn, A new approach to synthesis of porous SiO_x anode for Li-ion batteries via chemical etching of Si crystallites, *Electrochim. Acta* **2014**, 117, 426-430

[2] T. Fang, H. Liu, X. Luo, N. Gong, M. Sun, W. Peng, Y. Li, F. Zhang and X. Fan, Accommodation of two-dimensional SiO_x in a point-to-plane conductive network composed of graphene and nitrogen-doped carbon for robust lithium storage, *ACS Appl. Mater. Interfaces* **2022**, 14, 53658-53666

[3] K. Wang, Y. Tan, P. Li and J. Sun, Scalable 3D porous residual Al-doped Si/SiO_x composites for high performance anodes: Coupling effects of porosity, conductive sites and oxide layer, *Electrochim. Acta* **2020**, 353, 136538

[4] X. Sun, Z. Wang, H. Zhang, K. Si, X. Wang and X. Zhang, Honeycomb-like 3D ordered macroporous SiO_x/C nanoarchitectures with carbon coating for high-performance lithium storage, *J. Colloid Interface Sci.* **2023**, 651, 394-403

[5] J. Lee, J. Moon, S. A. Han, J. Kim, V. Malgras, Y.-U. Heo, H. Kim, S.-M. Lee, H. K. Liu and S. X. Dou, Everlasting living and breathing gyroid 3D network in Si@SiO_x/C nanoarchitecture for lithium ion battery, *ACS nano* **2019**, 13, 9607-9619

[6] X. Zhuang, P. Song, G. Chen, L. Shi, Y. Wu, X. Tao, H. Liu and D. Zhang, Coralloid-like nanostructured c-nSi/SiO_x@C_y anodes for high performance lithium ion battery, *ACS Appl. Mater. Interfaces* **2017**, 9, 28464-28472

[7] E. Park, M. S. Park, J. Lee, K. J. Kim, G. Jeong, J. H. Kim, Y. J. Kim and H. Kim, A highly resilient mesoporous SiO_x lithium storage material engineered by oil–water templating, *ChemSusChem* **2015**, 8, 688-694

[8] Y. Ling, Y. Gao, Y. Peng and S. Guan, Nitrogen-doped carbon-encapsulated ordered mesoporous SiO_x as anode for high-performance lithium-ion batteries, *Chemistry–An Asian Journal* **2022**, 17, e202200440

[9] Z. Liu, D. Guan, Q. Yu, L. Xu, Z. Zhuang, T. Zhu, D. Zhao, L. Zhou and L. Mai, Monodisperse and homogeneous SiO_x/C microspheres: A promising high-capacity and durable anode material for lithium-ion batteries, *Energy Storage Mater.* **2018**, 13, 112-118

[10] Z. Wang, L. Kong, Z. Guo, X. Zhang, X. Wang and X. Zhang, Bamboo-like SiO_x/C nanotubes with carbon coating as a durable and high-performance anode for lithium-ion battery, *Chem. Eng. J.* **2022**, 428, 131060

[11] T. Xu, Q. Wang, J. Zhang, X. Xie and B. Xia, Green synthesis of dual carbon conductive network-encapsulated hollow SiO_x spheres for superior lithium-ion batteries, *ACS Appl. Mater. Interfaces* **2019**, 11, 19959-19967

[12] H. Dong, J. Wang, H. Ding, P. Wang, R. Song, N. Zhang, F. Li and S. Li, The mosaic structure design to improve the anchoring strength of SiO_x@C@Graphite anode, *Mater. Today Chem.* **2021**, 22, 100599

[13] Z. Wang, N. Yang, L. Ren, X. Wang and X. Zhang, Core-shell structured SiO_x@C with controllable mesopores as anode materials for lithium-ion batteries, *Microporous Mesoporous Mater.* **2020**, 307, 110480

[14] T. Xiao, W. Zhang, T. Xu, J. Wu and M. Wei, Hollow SiO_2 microspheres coated with nitrogen doped carbon layer as an anode for high performance lithium-ion batteries, *Electrochim. Acta* **2019**, 306, 106-112

[15] Y. Jiang, D. Mu, S. Chen, B. Wu, Z. Zhao, Y. Wu, Z. Ding and F. Wu, Hollow silica spheres with facile carbon modification as an anode material for lithium-ion batteries, *J. Alloys Compd.* **2018**, 744, 7-14
